# Additive Effects of Exercise and Vitamin D Supplementation (with and without Calcium) on Bone Mineral Density in Older Adults: A Systematic Review and Meta-Analysis

**DOI:** 10.1155/2023/5570030

**Published:** 2023-08-08

**Authors:** Cecilie Fischer, Franz Jakob, Matthias Kohl, Stephanie Kast, Simon Von Stengel, Katharina Kerschan-Schindl, Uwe Lange, Friederike Thomasius, Stefan Peters, Michael Uder, Wolfgang Kemmler

**Affiliations:** ^1^Institute of Radiology, University Hospital Erlangen, Erlangen, Germany; ^2^Bernhard-Heine-Centrum für Bewegungsforschung, University of Würzburg, Würzburg, Germany; ^3^Department of Medical and Life Sciences, University of Furtwangen, Schwenningen, Germany; ^4^Austrian Society for Bone and Mineral Research, Vienna, Austria; ^5^German Society for Physical and Rehabilitative Medicine, Ulm, Germany; ^6^Osteology Umbrella Association Germany, Austria, Switzerland, Germany; ^7^German Association for Health-Related Fitness and Exercise Therapy (DVGS) e.V., Hürth-Efferen, Germany; ^8^Institute of Medical Physics, Friedrich-Alexander University of Erlangen-Nürnberg, Erlangen, Germany

## Abstract

Exercise is a recognized component in the prevention and therapy of osteoporosis. The present systematic review and meta-analysis aimed to determine the effect of Vitamin D (Vit-D) added to exercise versus exercise alone on bone mineral density (BMD) at the lumbar spine (LS) or hip in older adults. A systematic review based on six literature databases according to PRISMA included (a) exercise trials, with an exercise (EX) and a combined exercise + Vit-D group (EX + Vit-D), (b) intervention ≥ 6 months, and (c) BMD assessments at LS or hip. Effects sizes (MD) and 95%-confidence intervals (95%-CI) were calculated using a random-effect model that includes the inverse heterogeneity model (IVhet). Five studies with 281 participants in the EX and 279 participants in the EX + Vit-D were included. No significant differences between EX versus EX + Vit-D were observed for BMD-LS (MD: 0.002, 95%-CI: −0.033 to 0.036) or BMD-hip (MD: 0.003, 95%-CI: −0.035 to 0.042). Heterogeneity between the trial results was moderate-substantial for LS (*I*^2^ = 0%) and moderate for hip-BMD (*I*^2^ = 35%). The funnel plot analysis suggests evidence for a publication/small study bias for BMD-LS and hip results. In summary, this present systematic review and meta-analysis were unable to determine significant positive interaction of exercise and Vit-D on LS- or hip-BMD. We predominately attribute this finding to (1) the less bone-specific exercise protocols of at least two of the five studies and (2) the inclusion criteria of the studies that did not consequently focus on Vit-D deficiency. This issue should be addressed in more detail by adequately powered exercise trials with promising exercise protocols and participants with Vit-D deficiency. This trial is registered with the International Prospective Register of Systematic Reviews (PROSPERO) ID: CRD42022309813.

## 1. Introduction

Osteoporosis and corresponding fragility fractures are major problems in Western communities [1]. Due to the demographic change in Europe, the number of osteoporotic fractures is likely to increase by 25% during the next decade [1]. Physical exercise and Vit-D supplementation are considered as low threshold, cost-effective, and safely modifiable lifestyle factors [2] with relevant impact on bone health [3, 4] and fragility fracture reduction [5]. Although the mechanism of action of exercise and Vit-D on bone strength differs considerably, [6] studies have provided evidence for an interaction of Vit-D/Vit-D receptor (VDR) and exercise (i.e., mechanical loading) at the level of mechanotransduction [7, 8]. Correspondingly, there is some evidence that Vit-D supplementation might enhance the effects of exercise on bone mineral density (BMD) at least in people with Vit-D insufficiency–i.e., up to 50% of adults in Middle and Western Europe depending on 25(OH)D cut-off values [9]. In contrast to exercise, Vit-D supplementation can be considered as a low-effort intervention for addressing bone. Thus, considering potential interactions and the low burden of Vit-D supplementation, the effect of Vit-D supplementation added to exercise on bone is of relevance for nonpharmaceutic fracture prevention strategies. Thus, the aim of the systematic review and meta-analysis was to determine the effect of Vitamin-D (Vit-D) supplements added to physical exercise interventions (EX) on bone mineral density (BMD) at the lumbar spine (LS) and proximal femur in adults. We hypothesized that combined exercise + Vit-D interventions displayed significantly higher effects on BMD at the LS, or the total hip/femoral neck region of interest compared to an isolated exercise intervention.

## 2. Material and Methods

The literature search for this systematic review and meta-analysis followed the Preferred Reporting Items for Systematic Reviews and Meta-Analyses (PRISMA) Statement and was registered in the International Prospective Register of Systematic Reviews (PROSPERO; ID: CRD42022309813).

In the present study, we focus on literature searches of databases and registers only. Studies from the six electronic databases and registers (PubMed, Scopus, Ovid, Cochrane, Web of Science, and CINAHL) published up to 26th March 2023 were used for this review without language restrictions. A standard search strategy was developed using a standardized vocabulary (mesh term for MEDLINE; Table 1). To include all relevant studies, the following keywords and their synonyms were used: (“adults” OR “postmenopause” OR “post menopause” OR postmenopausal” OR “men”) AND (“Clinical trial” OR “Randomized clinical trial”) AND (“Exercise” OR “Training” OR “Athletic” OR “Sport” OR “physical activity”) AND (“Calcium” OR “Ca” OR “Vitamin D” OR “Milk” OR “Vit-D” OR “cholecalciferol”) AND (“Bone” OR “Bone metabolism” OR “Bone mineral content” OR “Bone Mineral Density” OR “BMD” OR “BMC”).

The keywords and their synonyms were combined to generate a search string that consisted of four segments, the trials (randomized controlled trials (RCT)), exercise, supplements, and BMD-part, as well as a combination of all four. The RCT-part of the search string was adjusted for some databases and registers according to current best practices (PubMed, Scopus, Ovid, and CINAHL). Even though some databases can be accessed via multiple options, e.g., MEDLINE via PubMed or Ovid, all six electronic databases and registers were still used to ensure that all relevant studies could be found. For each database and register, the RCT-part was used first, the exercise-part second, the supplement-part third, and the BMD-part fourth, and then, all four searches were combined. No further filters or limits were used during the search processes. The complete search returned 8086 results in total (1079 from PubMed, 4420 from Scopus, 895 from Ovid, 697 from Cochrane, 744 from Web of Science, and 251 from CINAHL). The reference lists of the five identified studies were manually reviewed, and a manual search of Google Scholar was performed to identify additional relevant articles. To exclude duplicate publications, author name, title, abstract, and date of publication were checked by the same reviewer (CF).

### 2.1. Eligibility Criteria

Studies/study arms with (1) randomized and nonrandomized controlled trial design with at least one exercise group without versus one exercise group with additional Vit-D supplementation, (2) ≥6 months intervention duration (shorter studies might not reach the full amount of mineralized bone and thus confound the BMD assessment), (3) areal BMD or BMC of the lumbar spine (LS) and femoral neck (FN) at baseline and end of study as determined by (4) dual X-ray absorptiometry (DXA), dual photon absorptiometry (DPA), or quantitative computer tomography (QCT), and (5) adults 50 years and older were included.

Studies involving (1) drugs (e.g. bisphosphonates, denosumab, HRT, glucocorticoids), (2) diseases (e.g., Cushing syndrome, hyperthyreosis, rheumatoid arthritis, diabetes mellitus type I), (3) conditions (immobility, paresis) with relevant influence on bone metabolism, (4) animal studies, (5) physical training using whole-body vibration (WBV) or electrical myostimulation (EMS), (6) review articles, case reports, editorials, conference abstracts, and letters, (7) studies that reported results of the same intervention (and no new/additional results of the interventions) were excluded.

### 2.2. Data Management

Search results were downloaded and imported to EndNote. Duplicates were identified and excluded based on the method proposed by Bramer et al. [10] Title and abstract screening as well as full-text screening was conducted using EndNote.

### 2.3. Selection Process

Titles and abstracts were screened by two independent reviewers (CF and SK). Disagreements were solved by discussion or with the help of a third reviewer (WK). The full-text articles of the relevant studies were also independently analyzed by two reviewers (CF and SK), and data were extracted from the included studies. The reason for excluding ineligible studies was also recorded. In case of disagreement and lack of consensus, a third reviewer (WK) made the decision.

### 2.4. Data Items

An extraction form applied in former studies [4, 11, 12] that concentrated on exercise effects on BMD was used to include relevant data. One author (CF) extracted the study, participant, and intervention characteristics, and two other authors (WK and SvS) checked and confirmed the results. In summary, publication characteristics (including first author, year of publication), study details (including sample size, dropout rate), participant characteristics (including health status, basal BMD/BMC values, age, height, weight, BMI, medication) (Table 2), exercise training characteristics (Table 3) (e.g., training status, training design, monitoring of training, intervention duration, type of exercise, intensity progression, attendance rate, specificity of exercise), and Vit-D supplement characteristics (Table 4) were tabulated. Data extraction was conducted using Microsoft Excel.

### 2.5. Outcome Measures

The outcome measure was BMD at the lumbar spine region of interest (ROI) determined by dual X-ray absorptiometry (DXA), dual photon absorptiometry, and quantitative computed tomography (QCT) as well as total hip or femoral neck BMD as determined by DXA or DPA. QCT results for the trabecular BMD at the LS [13] were not considered. Results for baseline BMD and the follow-up assessment immediately after the end of the intervention (or corresponding changes) were included in the analysis. Intermediate BMD results (e.g., 12-month BMD data of an 18-month intervention study [13]) were not considered.

### 2.6. Quality Assessment

Eligible studies were assessed for risk of bias by two independent reviewers (CF and WK) using the Physiotherapy Evidence Database (PEDro) scale risk of bias tool [14] and the Tool for the assEssment of Study qualiTy and reporting in EXercise (TESTEX) score [15] specifically dedicated to physiotherapy and/or exercise studies. In case of inconsistencies, a third independent reviewer (SK) made the decision.

### 2.7. Data Synthesis

Missing standard deviations (SD) were calculated using the method detailed in the recently published comprehensive meta-analysis by Shojaa et al. [4] In summary, standard errors (SE) [13, 16] and confidence interval (CI) [17] were converted to SD. [18] In the case of Mason et al. [19], who did not report any measure for the variation of change, we imputed the SD by using the correlations between baseline and final values from the other studies [18].

For those studies which measured BMD at multiple times, only the baseline and final values immediately determined after the intervention end was included in the analysis.

### 2.8. Statistical Analysis

We applied a random-effects meta-analysis using the metafor package [20] that is included in the statistical software R [21]. Effect size (ES) values were presented as mean differences (MDs) combined with the 95% confidence interval (95% CI). As a primary analysis, we performed a meta-analysis applying the robust inverse heterogeneity (IVhet) model [22]. Heterogeneity between studies was assessed using the *Q* and *I*^2^ statistic. An *I*^2^ of 0–40% is considered “low,” 30–60% is considered “moderate,” 50–90% is considered “substantial,” and 75–100% is considered “substantial heterogeneity” [23]. In addition to funnel plots, regression tests, and rank correlation effect estimates and their standard errors using the *t*-test and Kendall's *τ*-statistic for possible publication BIAS, we performed a trim-and-fill analysis using the L0 estimator proposed by Duval and Tweedie [24] In addition, we used DOI plots, the Luis Furuya-Kanamori index (LFK index), regression and rank correlation tests to check for asymmetry [25]. A *p* value <0.05 was used as the significance level for all tests.

### 2.9. Sensitivity and Subgroup Analysis

We excluded the study of Nelson et al. [16] from the sensitivity analysis due to the low dose of Vit-D (i.e., 280 IU/d) supplemented and the nonrandomized study design. Other sensitivity analyses focus on the varying effects of imputation strategy, i.e., the effect of imputing minimum correlation (maximum SD) or maximum correlation (minimum SD) in the case of Mason et al. [19]. Of importance for the main analysis, we used the result obtained by applying the mean of these correlations. Subgroup analyses were conducted to determine the potential confounding effects of additional Ca supplementation. We divided the studies into two categories: exercise EX + Vit-D only versus EX + Vit-D + Calcium supplementation.

## 3. Results

### 3.1. Study Characteristics

**Figure 1:**
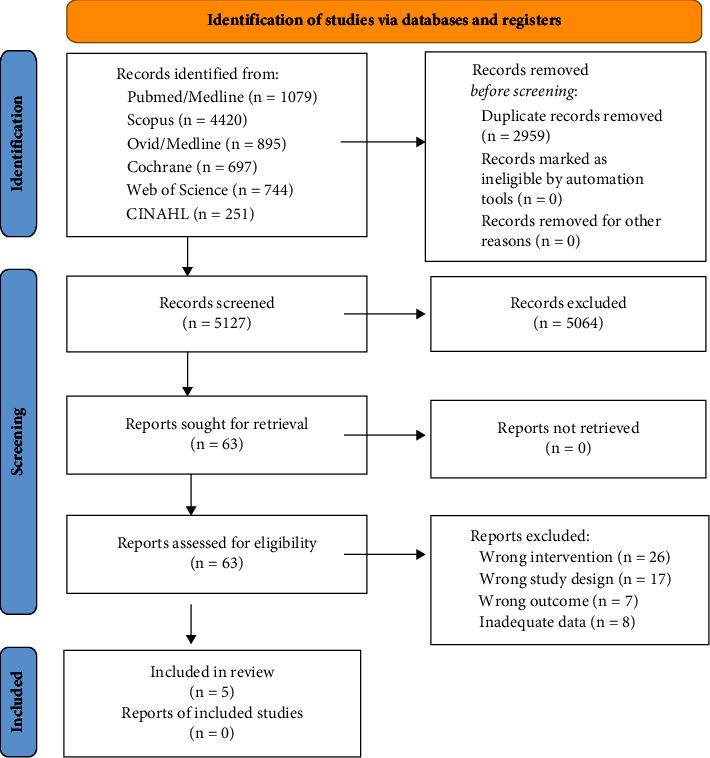
Flow diagram of the search process according to PRISMA [27].

Our literature search identified five eligible studies [13, 16, 17, 19, 26] (Figure 1), with five isolated exercises and five combined exercise/Vit-D study arms each. Three studies were randomized controlled trials, [13, 17, 19] while two studies applied a nonrandomized study design [16, 26]. The pooled number of included participants was 279 in the exercise and 281 in the combined group. Sample size of the study arms ranged from 9 [16, 26] to 109 [19] participants/group (Table 2). The studies were conducted in Australia [13], Finland [17], Spain [26], and the USA [16, 19] between 1991 [16] and 2022 [26].

### 3.2. Participant Characteristics

Table 2 reports the characteristics of the study participants. One study focused on older Caucasian men while the remaining four studies included postmenopausal predominately Caucasian women. All studies included healthy volunteers; one study particularly addressed overweight (BMI > 25 kg/m^2^) women. One study particularly focused on cohorts with osteopenia or osteoporosis [26]. All studies excluded participants under pharmaceutic therapy with an impact on calcium or bone metabolism.

### 3.3. Intervention Characteristics

#### 3.3.1. Exercise Characteristics

Table 3 displays the exercise characteristics of the trials. Of importance, with the possible exception of Garcia-Gomariz et al. [26] that did not report the exercise status of their cohort, all studies focus on physically less active participants or at least excluded persons [13, 17] with exercise habits with potential impact on bone. The intervention of three studies [13, 16, 26] focused on bone (-strength), one study determined effects on lean mass muscle strength and BMD [19] and another study addressed the number of falls as the primary outcome [17]. Two studies applied 24-month study interventions [17, 26], and the duration of the intervention of the three remaining studies was 12 months. The type of exercise varied considerably among the trials: three studies applied a weight-bearing and dynamic resistance exercise protocol [13, 17, 26]. One study focused on walking with weighted vests [16], and another study scheduled aerobic weight-bearing and nonweight-bearing exercises [19]. The attendance rate reported by all the authors showed that net training frequency ranged from two [13] to six sessions/week [17]. However, the latter protocol prescribed a daily home training of only 5–15 min. Thus, net training volume averages approximately between two [13] and three [16] hours per week. It is difficult to classify the bone-specific exercise intensity of the studies. Kukuljan et al. [13] applied high-impact weight bearing (up to 9.7x body mass) and high-intensity resistance (up to 85% 1RM) exercise while Nelson et al. and Manson et al. scheduled moderate-high intensity aerobic exercise protocols with [16] or without [19] weighted vests (Table 3). Unfortunately, Uusi-Rasi et al. [17] reported insufficient details of the exercise programs to estimate the bone-specific impact for its female cohort 70–80 years old. The same is true for the DRT sequence of Garcia-Gomariz et al. [26].

### 3.4. Vitamin D Characteristics

Table 4 gives the characteristics of Vit-D supplementation. No study implemented more than one Vitamin-D/exercise subgroup with respect to application or dose. As reported by four studies, the total baseline Vit-D intake of the cohorts ranged from 32 to 416 IU/d. Baseline 25(OH)D levels ranged from 21 to 36 ng/ml (Table 4). Vit-D supplementation of the five studies ranged from 2000 IU [19] to 284 IU/d [16]. The latter study focuses predominately on Ca supplementation; however, the “fortified” milk drink offered contained 284 IU/d. Unfortunately, cholecalciferol-induced changes of 25(OH)D levels were not listed by Garcia-Gomariz et al. [26] and Nelson et al., [16]. Kukuljan et al., [13] Manson et al. [19], and Uusi-Rasi et al. [17] reported 25(OH)D increases of about 30 [13] to 60% [19] in their EX + Vit-D subgroups–albeit with considerable individual variation. Apart from Nelson et al. [16], two other studies [13, 26] also supplemented calcium. Since prestudy Ca intake (850–1050 mg/d) was already in line with recent recommendations [28], we included these studies. Nevertheless, the effect of the additional calcium supplementation was analyzed in two subgroup analyses on LS and hip/femoral neck BMD.

### 3.5. Study Outcomes

All the studies determined areal BMD at the LS and femoral neck ROI using DXA or DPA. [16] Kukuljan et al. [13] also applied the QCT technique and determined volumetric BMD at the lumbar spine (not included in the present analysis).

### 3.6. Methodological Quality

Table 5 shows the methodological quality of the included studies according to the PEDro [14] and TESTEX score [15]. Following PEDro and applying the classification of Ribeiro de Avila et al. [30], the overall study methodological quality of the studies can be considered as high (PEDro ≥ 7 points}). However, in particular, aspects related to blinding were not satisfied or not reported. With respect to TESTEX, the nonreporting of adverse effects, and activity exercise monitoring outside the study protocol weaken the methodologic study quality of the studies. The training characteristics of all but one study were adequately described and can be reproduced by other researchers (Table 5).

Due to the aspect that not all studies [19] implemented a nontraining control group, it is difficult to determine the overall effect of exercise in the exercise or EX + Vit‐D groups. Kukuljan et al. [13] reported more favorable BMD data for the EG compared to the nontraining controls with and without supplements; however, changes after 12 months were more pronounced compared to the 18-month effects addressed here. Nelson et al. [16] observed significantly higher BMD effects (compared to the sedentary group) at the LS and femoral neck ROI in the combined but not in the isolated exercise group. Uusi-Rasi et al. [17] did not determine any significant differences between exercise and control groups for BMD at the LS or femoral neck after 24 months of intervention.

### 3.7. Meta-Analysis Results

#### 3.7.1. Effect of Exercise versus EX + Vit-D on Lumbar Spine BMD

**Figure 2:**
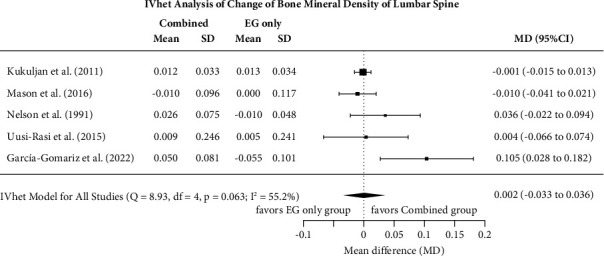
Forest plot of meta-analysis results at the lumbar spine. Data shown as pooled mean difference (MD) with 95% CI for changes in the combined exercise + Vit-D vs. isolated exercise groups.

Figure 2 displays the results of isolated exercise versus combined EX + Vit-D treatment on LS-BMD. In summary, the effect of combined EX + Vit-D on LS-BMD did not differ (*p*=0.912) from the effect of isolated exercise (MD: 0.002, 95%-CI: −0.033 to 0.036). Heterogeneity between the trials (*I*^2^ = 55%) was moderate-substantial (Figure 2). Sensitivity analysis with the exclusion of the study of Nelson et al. that applied very low Vit-D doses [16] did not relevantly affect (MD: −0.001, 95%-CI: −0.014 to 0.013) the effects size of the comprehensive analysis. Applying sensitivity analysis with respect to the imputation of the mean (see Figure 2), minimum correlation (maximum SD: MD: 0.002, 95%-CI: −0.025 to 0.029) or maximum correlation (minimum SD: MD: −0.004, 95%-CI: −0.034 to 0.026) displays consistently nonsignificant and roughly comparable effects.

The funnel plot with trim and fill analysis of the five included studies suggests evidence for a publication/small study bias. Two missing studies on the lower left-hand side (i.e., small studies with negative outcomes) were imputed, but this had no relevant influence on the overall effect (MD: −0.002, 95%-CI: −0.047 to 0.043) after adjusting for the missing study. The LFK index (4.21) confirmed this major asymmetry; however, the regression (*p*=0.22) and the rank correlation test (*p*=0.083) did not indicate significant funnel plot asymmetry.

#### 3.7.2. Subgroup Analysis on LS-BMD

In order to determine the potential confounding effects of additional Ca supplementation, we classified the studies into two categories. In summary, the effect of additional Ca in the combined EX + Vit-D group did not significantly (*p*=0.92) differ from the results for LS-BMD of the subgroup without additional calcium supplementation. Of importance, heterogeneity between the trial results of the subgroups (*I*^2^ = 32% for BMD-LS and *I*^2^ = 75%) was low and substantial, respectively.

#### 3.7.3. Effect of Exercise versus EX + Vit-D on Hip/Femoral Neck BMD

**Figure 3:**
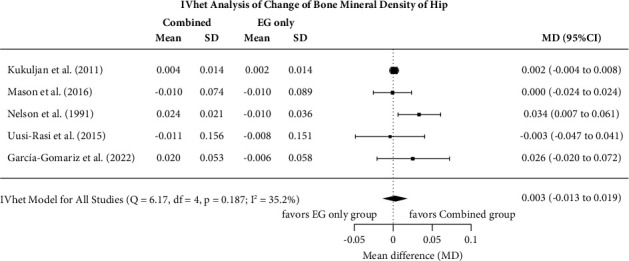
Forest plot of meta-analysis results at the hip region of interest. Data shown as pooled mean difference (MD) with 95% CI for changes in the combined exercise + Vit-D vs. isolated exercise groups.

In summary, the IVhet model (MD: 0.003, 95%-CI: −0.013 to 0.019) revealed no significant difference (*p*=0.675) between EX + Vit-D versus EX alone on hip/femoral neck BMD (Figure 3). Levels of heterogeneity between the trials can be considered low-moderate (*I*^2^ = 35%, Figure 3).

Sensitivity analysis determined similar effects each when imputing minimum (or maximum SD) or maximum correlation (minimum SD). Excluding the study of Nelson et al. [16] from the analysis did not relevantly change the result (MD: 0.002, 95%-CI: −0.004 to 0.008).

The funnel plot with trim and fill suggests evidence for a publication/small study bias. One missing study on the lower left-hand side (i.e., small studies with negative outcomes) was imputed, but this had no relevant influence on the overall effect (MD: 0.002; −0.021 to 0.025). The LKF index (2.76), however, not the regression (*p*=0.340) and the rank (*p*=0.483) correlation tests confirmed the results observed in the funnel plot.

#### 3.7.4. Subgroup Analysis on Hip-BMD

In summary, none of the subgroups, be it with or without additional calcium, revealed significant effects on the hip-BMD. Although MD of the combined EX + Vit-D subgroup with additional calcium was slightly higher (MD: 0.003, 95%-CI: −0.035 to 0.042) compared to the EX + Vit-D without Ca subgroup (MD: 0.004, 95%-CI: −0.015 to 0.023), the two categories did not differ significantly (0 = 0.976). This result predominately reflects the high variance and corresponding substantial heterogeneity (*I*^2^ = 80%) between the trial results of the “with Ca-subgroup,” while no heterogeneity (0%) was observed in the EX + Vit-D group.

## 4. Discussion

In summary, the present systematic review and meta-analysis do not indicate evidence for a superior effect of Vitamin D added to exercise versus exercise alone on BMD, be it at the LS or at the hip/femoral neck ROI in Vit-D sufficient participants. Thus, there is some evidence to revise our hypothesis of significantly more favorable BMD changes in the combined EX + Vit-D group compared to the exercise-only group. However, the evidence for this result is clearly limited by the specific participant and intervention characteristics of the few studies (Tables 2–4). Our result does not deny the usefulness of a combined intervention of regular physical exercise training and Vit-D in the area of fracture reduction. Apart from bone density, physical training [31] and Vit-D [32] are indisputably an obligatory component of osteoporosis therapy in the domain of falls, especially for people with a high risk of falling.

To our surprise, the number of exercise trials [13, 16, 17, 19, 33] that address the issue of additive effects of Vit-D on exercise was rather limited. Furthermore, some studies included after intensive discussions within the review team are not ideally suited to allow a straightforward and meaningful (meta-)analysis either. Aside from additional Ca supplementation [13, 16, 33], high variation of cholecalciferol supplementation (2000 IU/d [19] to 280 IU/d [16] (Table 4) and a study determining the effect of EX + Vit-D on BMD during weight loss, [19], the most confounding effect on our finding might be that at least (Uusi-Rasi et al. do not fully comprehensibly report their exercise protocol, thus we are unable to decide the osteogenic response of their exercise program) two of the five studies [16, 19] applied less bone-specific exercise protocols [34, 35]. This estimation was supported by the finding that, as reported above, Nelson et al. [16] and Uusi-Rasi et al. [17] failed to determine positive overall exercise effects for LS or femoral neck BMD compared to their nontraining controls.

Huge research programs on Vit-D and calcium supplementation and its impact on various outcomes such as bone health, falls, cancer, and respiratory infections have been conducted in the new millennium. It is an international consensus that there is a high incidence of Vit-D deficiency in Europe and Middle Eastern countries although there is no formal consensus on the exact definition of thresholds for deficiency. The European Calcified Tissue Society (ECTS) in a recent statement defined a serum level of 20 ng/ml of 25-OH vitamin D3 as the threshold for insufficiency [9]. This is of relevance when interpreting the results of the present meta-analysis because even the group with the lowest Vit-D levels (data for [26] not available) was in averge slightly above this level of insufficiency for bone health. Hence, the conclusion would be that additional supplementation has no significant additional effect on the efficacy of exercise on BMD accrual and maintenance in participants who are largely Vit-D sufficient or replete. This is supported by results of the DO-HEALTH program performed by Heike Bischoff-Ferrari and her group, where exercise and additional Vit-D supplementation in vitamin D-replete subjects showed no significant effects on bone and muscle parameters, while effects on the prevention of cancer and respiratory infections were seen in participants at risk [36–38], however. Having stated this, our results do not undermine the recommendations on Vit-D supplementation in persons at risk or of higher age (as defined >65 years of age) since it is of major importance to avoid Vit-D insufficiency in these populations for reasons of musculoskeletal and general health.

There is solid preclinical and clinical evidence for an interaction between exercise and the Vit-D/parathyroid hormone endocrine system. This is valid for the genomic and epigenomic actions of Vit-D/Vit-D receptor (VDR) and mechanical loading in preclinical settings and in vitro [7, 8, 39], as well as both during evolution and in modern societies [38, 40, 41]. Suggesting this direct or indirect (via respective target genes) permissive or even threshold-lowering effect of Vit‐D, highlights the relevance of adequate Vit‐D levels in exercise studies, but also the necessity to focus on patients at risk for Vit‐D supplementation.

Taking these limitations together, one might argue that a combined quantitative analysis of the data could not provide meaningful results. Retrospectively, we partially agree, nevertheless despite considerable differences between the studies (Tables 2–4), heterogeneity of trial results was negligible for LS-BMD (*I*^2^ = 0%) and low-moderate for the hip/femoral neck ROI.

Although most study limitations and features have been already addressed, some aspects should be still discussed. (1) Our search of eligible studies focused on databases and registers. One may argue that this approach might not identify all eligible reports or studies. However, one should bear in mind that this study was conducted within the framework of the (German) National guideline of fracture prevention that is based on several systematic reviews and meta-analyses in the area of exercise and BMD (e.g., [4, 11, 12, 42]). Using synergy effects between the searches and considering the close interaction between the researchers, we are very confident that all eligible studies have been included in the present work. (2) Due to the aspect that we observed relevant heterogeneity among the studies in a number of meta-analyses on training studies, [11, 43] we performed a random-effects meta-analysis and specifically chose the applied inverse heterogeneity model (IVhet) [22]. This model is less prone to underestimating the statistical error and thus leads to confidence intervals that meet the specified coverage probability better [44]. (3) Due to the limited number of studies included in the analysis (*n* = 4), the statistical power to clearly identify publication/small study bias was low. Thus, the result of the analyses should be interpreted carefully. (4) In some cases (Table 5), the TESTEX [15] score in particular revealed limitations in reporting the exercise protocol in adequate detail. This is essential, however, for interpreting the study results. (5) The duration of the intervention of all studies (≥12 months) was long enough to determine the full amount of mineralized bone [45, 46] and thus to provide reliable results. (6) Our research focuses exclusively on bone. Other pathologies, such as breast and colon cancer [47], autoimmune diseases [48], or diabetes [49], might benefit from earlier supplementation. Therefore, our results should not be interpreted as not to supplement subjects with a value of 20 ng/ml, which may be adequate for bone and muscle health, but not for the rest of the body.

## 5. Conclusion

In summary, the present systematic review and meta-analysis were unable to determine the significant positive interaction of exercise and Vit-D on LS- or hip-BMD. We predominately attribute this finding to two reasons: (1) the less bone-specific exercise protocols of at least two of five studies and (2) the inclusion criteria of the studies that did not consequently focus on Vit-D deficiency. In the future, well-designed, randomized control exercise trials should revisit this issue. Considering the low thresholds, cost-effectiveness, rare side effects, and multitarget potential of this approach, a sophisticated combination of exercise and Vit-D not only would be attractive but particularly for bone health in older people.

## Figures and Tables

**Table 1:** Example search using Cochrane library.

Search number	Query
24	#6 AND #11 AND #19 AND #23
23	#20 OR #21 OR #22
22	(Bone mass): ti, ab OR (bone mineral content): ti, ab OR (BMC): ti, ab OR (bone mineral density): ti, ab OR (BMD): ti, ab OR (bone metabolsim): ti, ab OR (bone density): ti, ab OR (bone turnover): ti, ab OR (bone formation): ti, ab OR (bone resorption): ti, ab OR (bone loss): ti, ab OR (bone strength): ti, ab OR (osteoporo^*∗*^): ti, ab OR (osteopenia): ti, ab
21	MeSH descriptor: [osteoporosis] explode all trees
20	MeSH descriptor: [bone density] explode all trees
19	#12 OR #13 OR #14 OR #15 OR #16 OR #17 OR #18
18	(Calcium supplement): ti, ab OR (vitamin d): ti, ab OR (vit-d):ti, ab OR (vitamin d supplement^*∗*^): ti, ab OR (dietary supplement^*∗*^): ti, ab OR (milk): ti, ab OR (nutrition): ti, ab OR (calciol dairy): ti, ab
17	MeSH descriptor: [calcium gluconate] explode all trees
16	MeSH descriptor: [calcium carbonate] explode all trees
15	MeSH descriptor: [calcium, dietary] explode all trees
14	MeSH descriptor: [ergocalciferols] explode all trees
13	MeSH descriptor: [cholecalciferol] explode all trees
12	MeSH descriptor: [Calcium] explode all trees
11	#7 OR #8 OR #9 OR #10
10	(Weight-bearing exercise): ti, ab OR (resistance training): ti, ab OR (fitness activit^*∗*^): ti, ab OR (physical activit^*∗*^): ti, ab OR (whole-body vibration): ti, ab OR (strength training): ti, ab OR (weight training): ti, ab OR (combat training): ti, ab OR (weight lifting): ti, ab OR (walking): ti, ab OR (aerobic training): ti, ab OR (aerobic exercise): ti, ab
9	MeSH descriptor: [vibration] explode all trees
8	MeSH descriptor: [sports] explode all trees
7	MeSH descriptor: [exercise] explode all trees
6	(#1 OR #2) NOT #5
5	#3 NOT #4
4	MeSH descriptor: [humans] explode all trees
3	MeSH descriptor: [animals] explode all trees
2	MeSH descriptor: [drug therapy] explode all trees
1	(Randomized controlled trial): pt OR (controlled clinical trial): pt OR (randomized): ti, ab OR (placebo): ti, ab OR (randomly): ti, ab OR (trial): ti, ab OR (groups): ti, ab

**Table 2:** Baseline characteristics of the studies (MV ± SD).

	Sample size		Gender	Age (years)	Body height (cm)	Body mass (kg)	Health status	Medication with impact on bone	Dropout (%)		Comments
	EX	EX + Vit-D							EX	EX + Vit-D	
Garcia-Gomariz et al. (2022)	9	16	w	>55	—	26 ± 2^a^	Osteopenia/osteoporosis	No	n.a	6	Design: EX vs. EX + Vit-D/Ca (vs. walking + Vit-D/Ca)
Kukuljan et al. (2011)	46	45	m	61 ± 7	174 ± 6	84 ± 11	Healthy	No	4	4	Design: EX vs. EX + Vit-D/Ca
Mason et al. (2016)	109	109	w	59 ± 5	n.g	87 ± 16	Overweight or obese	No	16	19	Design: EX vs. EX + Vit-D ...during weight loss program
Nelson et al. (1991)	12	9	w	60 ± 1	161 ± 1	64 ± 1	Healthy	No	25	0	Design: EX vs. EX + Vit-D/Ca (vs. Vit-D/Ca)
Uusi-Rasi et al. (2015)	103	102	w	74 ± 3	159 ± 6	72 ± 11	Healthy	No	12	6	Design: EX vs. EX + Vit-D (vs. Vit-D)

EX: exercise group; EX + Vit-D: exercise and Vit-D; m: men; n.a.: not applicable; n.g.: not given; w: women; ^a^BMI.

**Table 3:** Exercise characteristics of the studies.

First author (year)	Prestudy exercise status	Design, duration supervision	Main type(s) of exercise	Exercise composition per session	Progression of intensity	Attendance rate	BMD region of interest
Garcia-Gomariz el al. (2022)	n.g	Non-RCT, 24 months consistently-S	DRT, WB	3x 45 min/week; 10–15 min of walking and balance exercises, 30 min of high impact exercise (jumps) and DRT (free weights, elastic bands, etc.); no more details were given	n.g	>85% >85%	LS TH
Kukuljan et al. (2011)	No RT or HI-WB in last 6 months	RCT, 12 months, partially-S	DRT (focus on spine, hip muscles groups) and high-impact WB	3x week; linearly periodized DRT, up to 13 exercises, 2 sets 8–12 reps, one warm-up set at 60–65% 1RM, one set at 60–85% 1RM; 90–180 reps/session of stepping, jumping, landing with GRF 1.5–9.7x body weight	Yes	65% (EX) 69%	LS TH
Mason et al. (2016)	No recent exercise	RCT, 12 months, partially-S	Aerobic exercise (WB and non-WB)	5x 45 min/week walking, jogging, bicycling, and other aerobic machines 70–85% HRmax	Yes	56% (EX) 59%	LS FN
Nelson et al. (1991)	Sedentary	Non-RCT, 12 months, consistently-S	Walking with weighted vests	4x 50 min/week walking with a 3.1 kg weighted vest at 75–80% HRmax	No	>90%	LS FN
Uusi-Rasi et al. (2015)	No mod.- intense EX > 2 h/week	RCT, 24 months, partially-S	DRT, WB, balance, and mobility	1^a^-2x week group-EX and daily HE (5–15 min). DRT: ?exercises, ?sets, ?reps at up to 75% 1RM, no details on WB, balance, and mobility exercises were given	Yes	73% (GE) 66% (HE)	LS FN

^a^Last 12 months; DRT: dynamic resistance exercise; EX: exercise; FN: femoral neck; GRF: ground reaction forces; HE: home exercise training; LS: lumbar spine; reps: repetitions; RT: resistance training; S: supervised; TH: total hip; WB: weight bearing.

**Table 4:** Vit-D and Ca supplement characteristics of the included studies (MV ± SD).

Study	Baseline intake Vit-D (IU/d)	Baseline 25(OH)D-levels (ng/ml)	Vit-D supplementation (IU/d)	Baseline intake calcium (mg/d)	Ca supplementation (mg/d)
Garcia-Gomariz et al. (2022)	EX: n.g	n.g	400	n.g	600
	Comb: n.g	n.g		n.g	

Kukuljan et al. (2011)	EX: 32 ± 44^a^	34 ± 16	800	911 ± 360	1000
	Comb: 48 ± 84	36 ± 12		1064 ± 449	

Mason et al. (2016)	EX: 580^b^	21 ± 6	2000	1170 ± 633	—
	Comb: 515	21 ± 6		1071 ± 564	

Nelson et al. (1991)	EX: 116 ± 60^a^	30 ± 10	284	869 ± 228	831
	Comb: 140 ± 96	28 ± 13		889 ± 303	

Uusi-Rasi et al. (2015)	EX: 412 ± 144	28 ± 7	800	1119 ± 346	—
	Comb: 416 ± 156	26 ± 7		1109 ± 385	

^a^dietary vitamin D intake only, ^b^baseline dietary vitamin D intake and supplement intake were added.

**Table 5:** Methodologic quality of the included trials (*n* = 5) applying the PEDro [29] and TESTEX [15] score.

Author, year	PEDro criteria												Additional TESTEX criteria^1^					
	Eligibility criteria	Random allocation^2^	Allocation concealment	Intergroup homogeneity	Blinding subjects	Blinding personnel	Blinding assessors	Participation ≥ 85% allocation	Intention to treat analysis^3^	Between-groupcomparison	Measure of variability	Total score PEDro	Adverse effects reported	Attendance reported	Activity monitoring in control groups	Relative exercise intensity constant	Exercise volume & energy expended	Total score TESTEX
García-Gomariz et al. 2022	Yes	—	—	+	—	+	+	+^4^	+	+	+	**7**	+	−^4^	+	—	+	**10**
Kukuljan et al. 2011	Yes	+	+	+	—	—	—	+	+	+	+	**7**	+	+	—	+	+	**13**
Mason et al. 2016	Yes	+	+	+	+	+	—	—	+	+	+	**8**	+	+	—	+	+	**12**
Nelson et al. 1991	Yes	—	—	+	+	+	—	+	+	+	+	**7**	—	+	—	+	+	**10**
Uusi-Rasi et al. 2015	Yes	+	+	+	—	—	+	+	+	+	+	**8**	+	+	—	+	—	**13**

^1^TESTEX awards one point for listing the eligibility criteria and, also in contrast to PEDro, a further point for the between-group comparison of at least one secondary outcome. Further, TESTEX did not address the blinding of study participants and personal/caregivers. Finally, “random allocation” as defined by TESTEX refers only to the patient/participant, ^2^computer-generated randomization methods were considered as “random allocation.” ^3^…. or all subjects received treatment or control as allocated...(TESTEX however focuses on the application of an ITT-analysis), ^4^…. data were provided by the authors upon request. The bold values indicate the total score points for the PEDro and TESTEX score.

## Data Availability

The datasets generated and/or analyzed during the current study are available from the corresponding author upon reasonable request.
